# Claudins are essential for cell shape changes and convergent extension movements during neural tube closure

**DOI:** 10.1016/j.ydbio.2017.05.013

**Published:** 2017-08-01

**Authors:** Amanda I. Baumholtz, Annie Simard, Evanthia Nikolopoulou, Marcus Oosenbrug, Michelle M. Collins, Anna Piontek, Gerd Krause, Jörg Piontek, Nicholas D.E. Greene, Aimee K. Ryan

**Affiliations:** a Department of Human Genetics, McGill University, Canada; b Department of Experimental Medicine, McGill University, Canada; c Department of Anatomy and Cell Biology, McGill University, Canada; d Department of Pediatrics, McGill University, Canada; e The Research Institute of the McGill University Health Centre, Montréal, Québec, Canada; f Developmental Biology and Cancer Programme, Birth Defects Research Centre, UCL Institute of Child Health, London, UK; g Leibniz-Forschungsinstitut für Molekulare Pharmakologie, FMP, Berlin, Germany; h Institute of Clinical Physiology, Charité-Universitätsmedizin Berlin, Berlin, Germany

**Keywords:** C-CPE, C-terminal domain of *Clostridium perfringens* enterotoxin, NTD, Neural tube defect, Claudin, Neural tube defects, Tight junctions, Apical constriction, Convergent extension

## Abstract

During neural tube closure, regulated changes at the level of individual cells are translated into large-scale morphogenetic movements to facilitate conversion of the flat neural plate into a closed tube. Throughout this process, the integrity of the neural epithelium is maintained via cell interactions through intercellular junctions, including apical tight junctions. Members of the claudin family of tight junction proteins regulate paracellular permeability, apical-basal cell polarity and link the tight junction to the actin cytoskeleton. Here, we show that claudins are essential for neural tube closure: the simultaneous removal of Cldn3, −4 and −8 from tight junctions caused folate-resistant open neural tube defects. Their removal did not affect cell type differentiation, neural ectoderm patterning nor overall apical-basal polarity. However, apical accumulation of Vangl2, RhoA, and pMLC were reduced, and Par3 and Cdc42 were mislocalized at the apical cell surface. Our data showed that claudins act upstream of planar cell polarity and RhoA/ROCK signaling to regulate cell intercalation and actin-myosin contraction, which are required for convergent extension and apical constriction during neural tube closure, respectively.

## Highlights

• Simultaneous removal of Cldn3, −4 and −8 causes open neural tube defects.
• Folic acid cannot rescue open NTDs caused by depletion of Cldn3, −4 and −8.
• Removal of Cldn3, −4 and −8 prevents convergent extension.
• Apical constriction to form the median hinge point requires Cldn3, −4 and −8.
• Claudins localize polarity complex components to the apical surface.

## Graphical abstract

**Figure:**
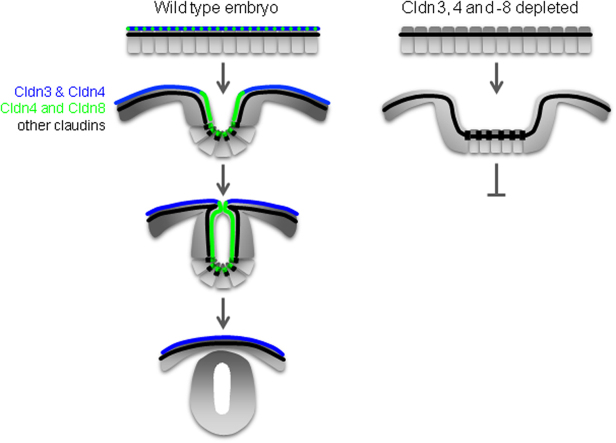

## 1 Introduction

The molecular interactions that regulate many of the morphogenetic changes required for neural tube closure occur at the apical cell surface (Colas and Schoenwolf, 2001, Lawson et al., 2001). Apical localization of Wnt/planar cell polarity (PCP) pathway components is required for the convergent extension movements that lead to anterior-posterior elongation and medial-lateral narrowing of the neural plate (Goto and Keller, 2002, Wallingford and Harland, 2002, Wang et al., 2006). Apical constriction of cells at the midline to form the median hinge point depends upon the actin-myosin contractile force driven by localized myosin light chain phosphorylation downstream of RhoA-ROCK signaling (Suzuki et al., 2012, Kinoshita et al., 2008). This allows the neural plate to bend and the neural folds to elevate. During the final phase of neural tube closure, Cdc42 RhoGTPase signaling regulates the formation of apical protrusions which are essential for the epithelial remodeling at the boundary of the neural and non-neural ectoderm to form the closed neural tube and a continuous layer of overlying surface ectoderm (Lawson et al., 2001, Pai et al., 2012, Moury and Schoenwolf, 1995, Quiros and Nusrat, 2014, Collins et al., 2013).

Apical tight junctions are a hallmark of vertebrate epithelial cell layers. They maintain apical-basal polarity by preventing the mixing of apical and basolateral membrane proteins and regulate the paracellular movement of ions, solutes and water (Gunzel and Yu, 2013, Suzuki et al., 2015). The claudin family of tetraspan membrane proteins are essential for formation of the tight junction backbone through homo- and heteropolymerization: their four transmembrane helix bundles interact within one cell membrane and their two extracellular loops interact with those of claudins in the apposing cell (Gunzel and Yu, 2013, Daugherty et al., 2007, Krause et al., 2015). While the claudin extracellular loops define the paracellular barrier properties of the junction, their cytoplasmic C-termini interact with adaptor and scaffolding protein complexes at the tight junction cytoplasmic plaque. Thus, claudins are poised at the apical surface to bridge intercellular interactions to cytoplasmic regulatory events that affect cell behaviours.

Our previous analysis of claudin expression patterns in chick embryos revealed that 14 claudins are expressed during neurulation (Collins et al., 2013). Neural tube defects have not been reported in any of the single claudin knockout mouse lines (Morita et al., 1999, Furuse et al., 2002, Gow et al., 1999, Ben-Yosef et al., 2003, Tamura et al., 2008, Miyamoto et al., 2005, Kage et al., 2014, Li et al., 2014, Fujita et al., 2012, Anderson et al., 2006). However, *Cldn4*, *−6*, and *−7* are downregulated in *Grhl2* mutant mouse lines, which exhibit open neural tube defects due to a failure in the final stage of neural tube closure (Rifat et al., 2010, Pyrgaki et al., 2011, Werth et al., 2010). These data suggest that claudins may have functionally redundant roles during neural tube closure.

The C-terminal domain of *C. perfringens* enterotoxin (C-CPE) is an ideal tool for studying claudin redundancy. Full-length CPE, a common cause of food poisoning, crosses the intestinal barrier at tight junctions by binding to the second extracellular loop of Cldn4, which was first cloned as the CPE cell-surface receptor (CPE-R) (Katahira et al., 1997a). CPE also interacts with Cldn3, −6, −7, −8, −9, −14 and −19 (Katahira et al., 1997, Winkler et al., 2009, Shrestha and McClane, 2013) but not with other cell surface proteins (Katahira et al., 1997, Lohrberg et al., 2009). C-CPE contains the claudin binding domain but not the cytotoxic domain, which is located in the N-terminal region of CPE. C-CPE can bind to and internalize the same subset of claudins without affecting the localization of other claudin family members (Winkler et al., 2009, Sonoda et al., 1999). C-CPE was used to demonstrate the redundant functions of Cldn4 and −6 in mouse blastocysts: mice null for only Cldn4 (Fujita et al., 2012) or only Cldn6 (Anderson et al., 2006) are viable but removal of both claudins by C-CPE caused a loss of the hydrostatic pressure that maintains blastocoel shape (Moriwaki et al., 2007).

Here, we determined that C-CPE-sensitive claudins are essential for neural tube morphogenesis. Targeted removal of Cldn3, −4 and −8 from the neural and non-neural ectoderm of neural plate stage chick embryos resulted in folate-resistant open neural tube defects (NTDs). We showed that C-CPE-sensitive claudins were required for convergent extension movements and apical constriction of cells at the median hinge point. Furthermore, our data suggest that claudin family members differentially regulate localization of components of the PCP polarity complex and RhoGTPase signaling to the apical cell surface.

## 2 Results

### 2.1 C-CPE-sensitive claudins are required for neural tube closure in chick embryos

**Fig. 1:**
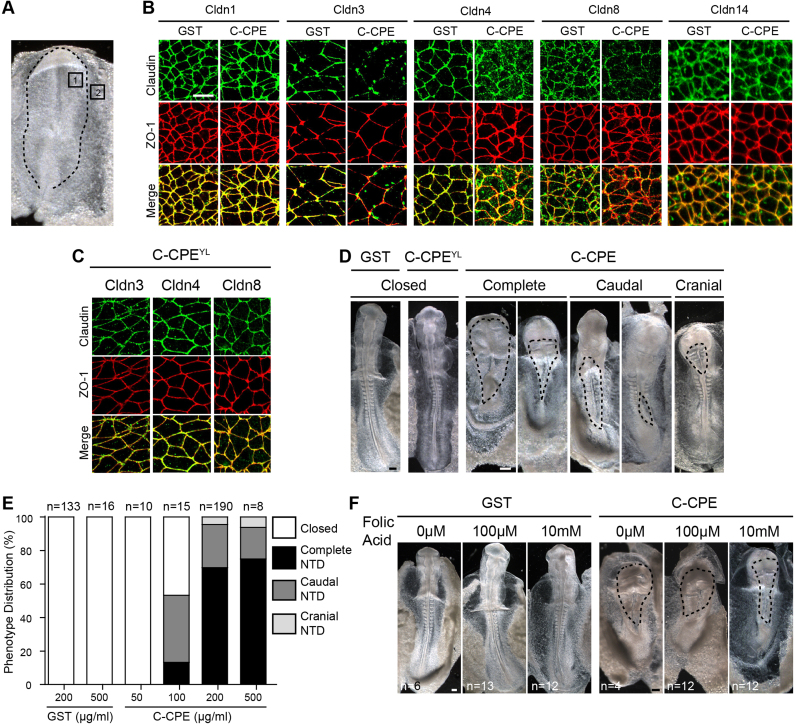
C-CPE-treated embryos exhibit dose-dependent, folic acid resistant neural tube defects. **(A)** Dorsal view of a neural groove stage embryo. Dashed line outlines the neural plate. The areas of the neural (box 1) and non-neural (box 2) ectoderm imaged in (B) are shown. **(B)** Apical surface view of ZO-1 (red) and Cldn1, −4, −8 or −14 (green) in the neural ectoderm and Cldn-3 (green) in non-neural ectoderm of 5 h GST- or C-CPE-treated embryos. Three embryos per treatment were analyzed. Scale bar, 10 µm. **(C)** Apical surface views of ZO-1 (red) and Cldn3, −4 or −8 (green) in embryos treated with C-CPE^YL^ for 5 h. Three embryos per treatment were analyzed. Scale bar, 10 µm. **(D)** Dorsal views of chick embryos treated with 200 µg/ml GST, C-CPE^YL^, or C-CPE for 20 h. Dashed lines indicate open neural tubes. Scale bar, 0.2 mm. **(E)** Distribution of complete, cranial and caudal open NTDs following 20 h incubation in 200 or 500 µg/ml GST or 50, 100, 200 or 500 μg/ml C-CPE. **(F)** Dorsal views of embryos treated with 200 µg/ml GST or C-CPE in the presence of 0 μM, 100 μM, or 1 mM folic acid. Dashed lines indicate open neural tubes. Scale bar, 0.2 mm.

**Suppl Fig. 1:**
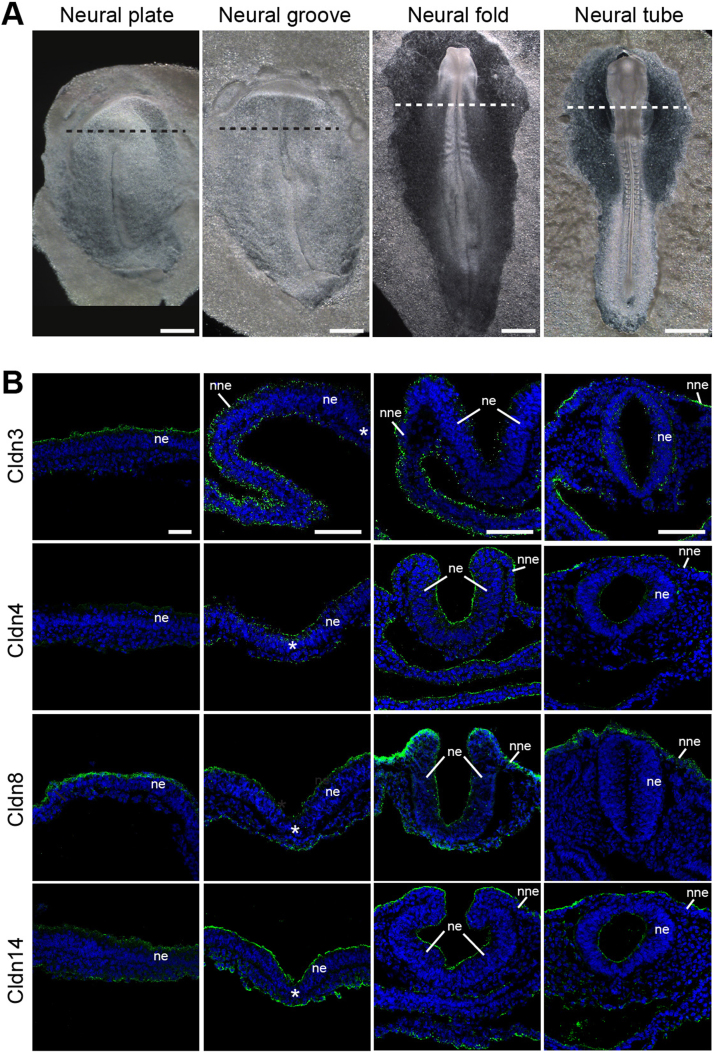
Expression of claudins at neurulation in chick embryos. **(A)** Dorsal views of chick embryos at the different stages of neural tube closure. Dashed lines indicate the level of sections shown in B. Scale bar, 0.5 mm. **(B)** Immunostaining for Cldn3, −4, −8 and −14 (green) on transverse sections of chick embryos during neurulation corresponding to the level of the dashed lines in A. Nuclei are stained with DAPI (blue). Asterisk indicates the midline. ne, neural ectoderm; nne, non-neural ectoderm. Scale bar, 100 µm.

To simultaneously remove multiple claudins from tight junctions during neurulation we used the nontoxic GST-C-CPE (hereafter referred to as C-CPE) reagent and compared its effects to either GST alone or C-CPE^YL^, a variant of C-CPE that does not bind claudins (Protze et al., 2015). Previously we showed by whole mount *in situ* hybridization that *Cldn3*, *−4*, *−8* and *−14* are the only C-CPE-sensitive claudins expressed during neural tube closure in chick embryos. We first confirmed that the protein expression patterns of these claudins during neural tube closure matched that of their transcripts (Collins et al., 2013). As expected Cldn4, −8 and −14 were expressed in the neural ectoderm, while Cldn3 was absent from the neural folds but was highly expressed in non-neural ectoderm (Supplementary Fig. 1). Next, we tested the ability of C-CPE to effectively remove these claudins from tight junctions as compared to effects on Cldn1, which does not interact with C-CPE (Fig. 1A and B). In GST-treated embryos, all five claudins co-localized with the tight junction scaffolding protein ZO-1 at apical cell-cell contacts in the neural (Cldn1, −4, −8, −14) and non-neural (Cldn1, −3, −4, −14) ectoderm (Fig. 1B). Co-localization analysis using Pearson's correlation coefficient confirmed that Cldn1 (R=0.6267), −3 (R=0.5583), −4 (R=0.5867), −8 (R=0.5070), and −14 (R=0.7156) co-localized at tight junctions with ZO-1, which was used as a marker of tight junctions. After 5 h of C-CPE treatment, only Cldn1 (R=0.6975) and Cldn14 (R=0.6083) remained co-localized with ZO-1 at tight junctions; localization of Cldn3 (R=0.09563), −4 (R=0.09) and −8 (R=0.2519) was discontinuous and often absent (Fig. 1B). Similar effects were observed after 20 h (data not shown). The unexpected observation that Cldn14 remained localized to tight junctions in C-CPE-treated embryos may reflect context-dependent sensitivity to C-CPE. As predicted, C-CPE^YL^ had no effect on the localization of Cldn3, --4 or --8 (Fig. 1C).

**Suppl. Fig. 2:**
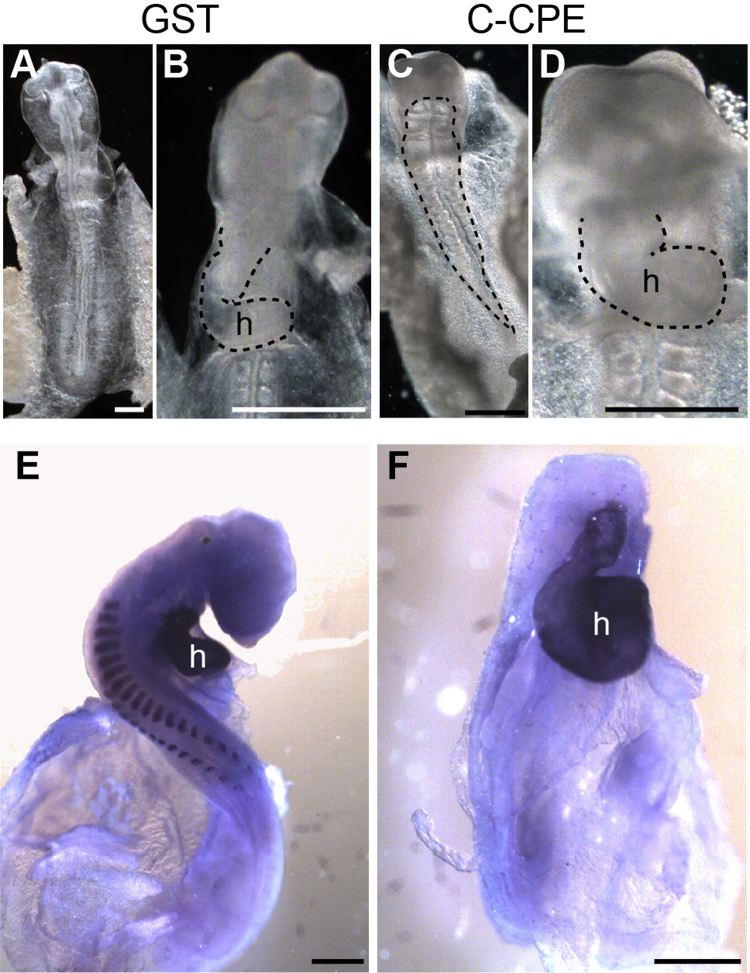
Embryos treated with C-CPE are viable. **(A, C)** Dorsal view of embryos treated with 200 µg/ml GST (A) or C-CPE (C) for 20 h. Scale bar, 0.5 mm. **(B, D)** Ventral view showing the rightwardly looped heart tube of embryos shown in A and C. h, heart. Scale bar, 0.5 mm. **(E, F)***Troponin* expression in embryos cultured in 200 µg/ml GST (E) or C-CPE (F) for 40 h. h, heart. Scale bar, 0.5 mm.

To determine if C-CPE-sensitive claudins are required for neural tube closure, HH4 neural plate stage embryos were cultured *ex ovo* in GST or in C-CPE media for 20 h. GST-treated embryos and embryos treated with the C-CPE^YL^ variant were indistinguishable from wild type embryos grown *in ovo* (Fig. 1D). C-CPE-treatment did not affect embryo viability: at 20 h their hearts were beating, of normal size and exhibited normal rightward looping (Movie 1; Supplementary Fig. 2). However, C-CPE-treated embryos showed a dose-dependent increase in the incidence of open NTDs (Fig. 1E). NTDs were characterized as ‘complete’ when the opening was along the entire anterior-posterior axis, ‘caudal’ when the opening was posterior to the hindbrain or ‘cranial’ when the opening was in the region of the future brain (Fig. 1D and E). The lowest dose of C-CPE that caused NTDs in 100% of embryos (200 μg/ml) was used for all subsequent experiments. Folic acid supplementation, which reduces the incidence of NTDs in humans by 60–70% (van der Linden et al., 2006) and rescues NTDs induced in chick embryos (Guney et al., 2003, Weil et al., 2004), was unable to rescue the NTDs in C-CPE-treated embryos (n=24; Fig. 1F), suggesting that the C-CPE-induced NTDs are a model of folate-resistant NTDs.

**Movie 1:**
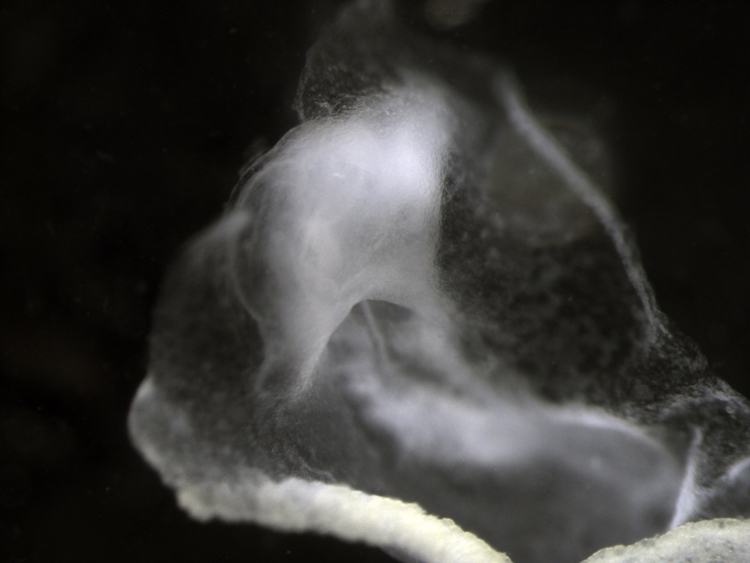
**The heart of C-CPE-treated embryos is beating.** Movie showing the beating heart of a C-CPE-treated embryo (left) cultured *ex ovo* using the Cornish Pasty method for 20 h. **Movie 1 Still.** Ventral view showing the rightwardly looped heart tube of a C-CPE-treated embryo.

### 2.2 Evolutionarily-conserved requirement for claudins in neural tube closure in mice

**Fig. 2:**
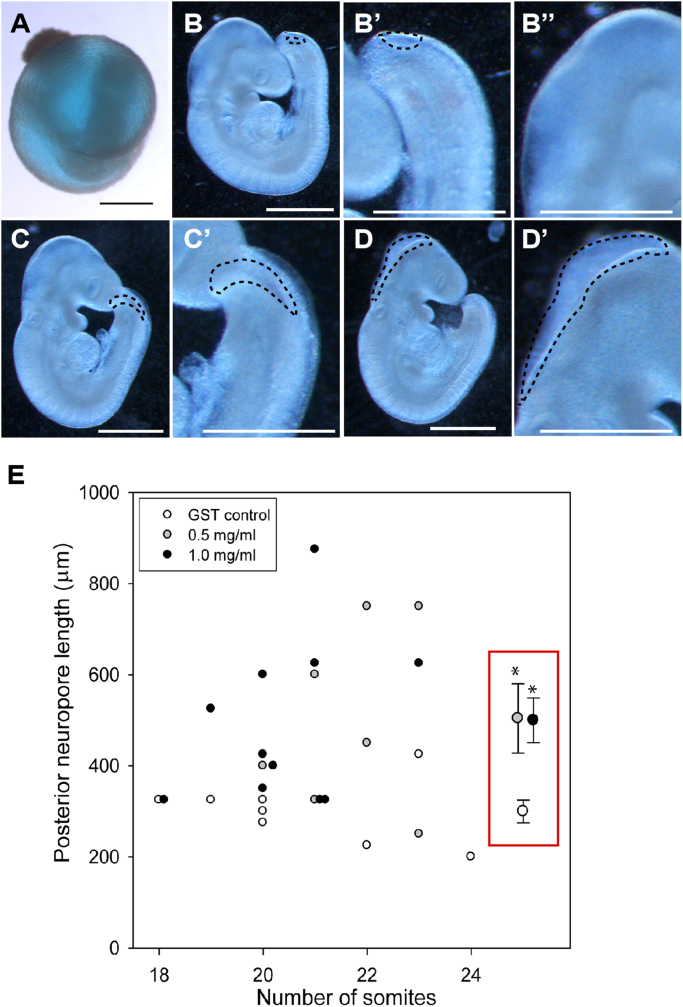
C-CPE prevents closure of the posterior neuropore in mouse embryos. **(A-D)** Cultured mouse embryos injected with GST or C-CPE into the amniotic cavity prior to culture. (**A**) Blue dye indicates retention of solution in yolk sac during 18–20 h culture period. (**B-D**) Representative embryos injected at E8.5 with (B) GST, (C) 0.5 mg/ml C-CPE or (D) 1.0 mg/ml C-CPE. Dashed lines indicate normal neuropore (B′) and closed neural tube (B″) in GST-treated embryos and enlarged neuropore in (C,C′) and open hindbrain neural folds (cranial NTD) in (D,D′) in C-CPE-treated embryos. **(E)** The length of the posterior neuropore relative to the somite number in individual embryos at the end of the culture period is shown. The mean (± s.e.m.) for each treatment group is shown in the red rectangle. (*p<0.025, ANOVA).

To determine if removal of C-CPE-sensitive claudins would affect neural tube closure in mouse embryos, C-CPE was injected into the amniotic cavity of embryos that were then cultured for approximately 18 h (Fig. 2A). Among embryos injected at the 0–4 somite stage, exposure to 0.5 or 1 mg/ml led to shortening of the caudal end of the embryo and a ‘wiggly’ neural tube suggestive of caudal elongation defects and a proportion of these embryos (n=4/7) also failed to undergo axial turning. As these embryos did not progress to a stage where neural tube closure could be reliably assessed due to other confounding phenotypes, we focused our analysis on embryos in which treatment was performed after initiation of neural tube closure at the 9–13 somite stage. Treated embryos appeared healthy and viable after the culture period; yolk sac circulation and mean number of somites in C-CPE-treated embryos did not significantly differ from controls. Spinal neural tube closure continued to progress in all treatment groups. However, the open region of spinal neural folds (posterior neuropore) was significantly larger in embryos treated with C-CPE (Fig. 2C,D) than in GST controls (Fig. 2B), indicating suppression of closure. Moreover, cranial NTDs were also observed in embryos exposed to 0.5 (n=1/8) and 1 mg/ml (n=3/12) C-CPE (Fig. 2D). These data indicate that C-CPE-sensitive claudins are also required for neural tube closure in mouse embryos. The ‘milder’ NTDs observed in the C-CPE-treated mouse embryos most likely reflects the fact that neural tube closure was already initiated at the time of treatment.

### 2.3 Depletion of C-CPE-sensitive claudins does not affect ectoderm differentiation

**Suppl. Fig. 3:**
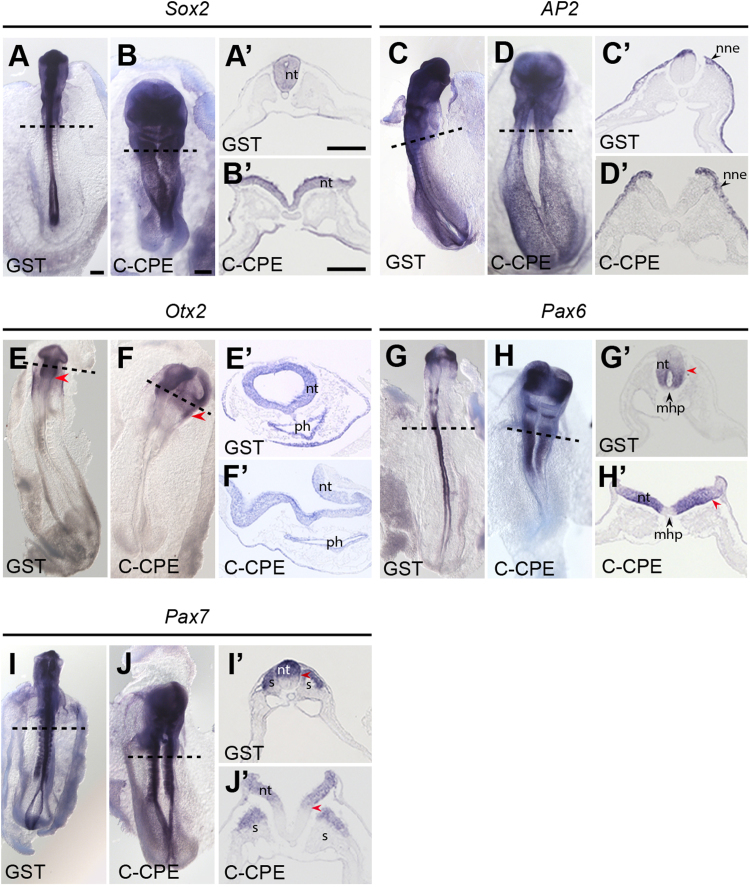
Differentiation and patterning of the neural and non-neural ectoderm were not affected by C-CPE treatment. Wholemount in situ hybridization of embryos treated with GST or C-CPE for 20 h with *Sox2*, which marks the neural tube and endoderm (A, B), *AP-2α*, which marks non-neural ectoderm (C, D), *Otx2*, which marks the anterior neural tube (E,F), *Pax6*, which marks the optic vesicles, the first rhombomere and the ventral neural tube (G,H), and *Pax7*, which marks the dorsal neural tube (I, J). A’-J’ are transverse sections through whole embryos at the level of the dashed line in A-J. Red arrowheads mark expression boundaries. mhp, median hinge point; ne, neural ectoderm; nne, non-neural ectoderm; nt, neural tube; ph, pharynx; s, somite. Scale bar, 0.2 mm.

**Suppl. Fig. 4:**
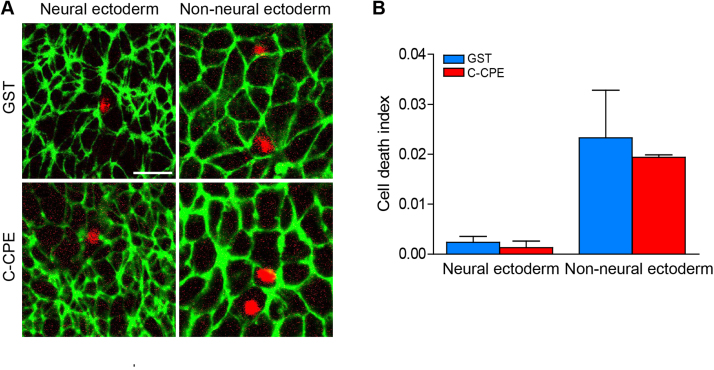
Embryos treated with C-CPE are viable and do not exhibit increased cell death. **(A)** Apical surface views of neural and non-neural ectoderm in embryos treated with GST or C-CPE for 5 h and assayed for apoptosis by TUNEL assay. TUNEL-positive nuclei are red. Phalloidin (green) outlines cells. Three embryos per treatment were analyzed. Scale bar, 10 µm. **(B)** Graph shows cell death index in the neural and non-neural ectoderm of embryos treated with GST or C-CPE for 5 h (mean±s.e.m, p > 0.05, student-*t*-test).

Differentiation of the ectoderm into neural and non-neural progenitors is the first step of neurulation and disrupting this process can affect neural tube closure (Ybot-Gonzalez et al., 2007, Kimura-Yoshida et al., 2015, Ybot-Gonzalez et al., 2007). To determine if the open neural tube defects caused by C-CPE-treatment were downstream of effects on cell type differentiation, we examined gene expression patterns in embryos treated with C-CPE for 20 h (Supplementary Fig. 3). Despite the clear morphological defects following C-CPE treatment, all genes examined exhibited spatially-restricted expression boundaries that were similar to those observed in GST-treated control embryos. *Sox2* was expressed in the neural ectoderm and *AP2* was expressed in the non-neural ectoderm indicating that the initial differentiation event that distinguishes neural versus non-neural progenitors occurred normally (Supplementary Fig. 3A-D). *Otx2* and *Pax6* exhibited normal boundaries of expression along the anterior-posterior axis (Supplementary Fig. 3E-H). Finally, differentiation of ventral versus dorsal cell types in the neural tube was also initiated normally in C-CPE-treated embryos based on the ventrally- and dorsally-restricted expression domains of *Pax6* and *Pax7*, respectively, in the open neural tubes (Supplementary Fig. 3G-J). Thus, failure of the neural tube to close in C-CPE-treated embryos is not due to a global defect in differentiation or patterning of the ectoderm. Furthermore, we did not observe a difference in cell death between GST- and C-CPE-treated embryos at 5 h and 20 h (p>0.05) (Supplementary Fig. 4 and data not shown).

**Fig. 3:**
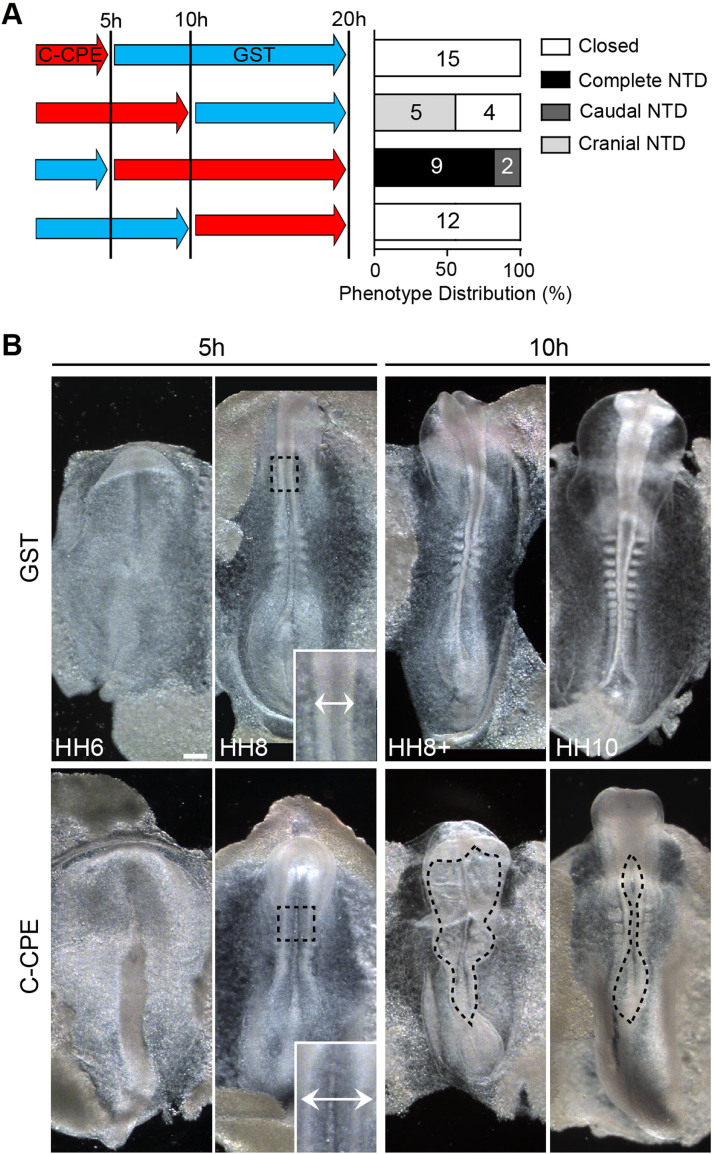
C-CPE-sensitive claudins are required for apposition of neural folds. **(A)** Time course of media swap (left) and phenotypes of neural tube defects at the end of the 20 h of culture period (right). Numbers of embryos per phenotype are indicated. **(B)** Dorsal views of embryos cultured from the neural plate stage (HH4) in 200 µg/ml GST or C-CPE for 5 h or 10 h. Double-headed arrows indicate distance between midbrain neural folds (inset). Dashed lines outline open NTDs. Scale bar, 0.2 mm.

### 2.4 Claudins are required during neural plate shaping and median hinge point formation

To identify the phases of neural tube closure dependent on C-CPE-sensitive claudins, we performed media swap experiments. C-CPE does not act at the level of claudin transcription or translation, therefore, upon its removal C-CPE-sensitive claudins are able to re-populate tight junctions (Sonoda et al., 1999). All embryos transferred from C-CPE to GST media at 5 h and collected at 20 h had closed neural tubes (Fig. 3A). However, ~50% of the embryos transferred from C-CPE to GST at 10 h had cranial NTDs. In a complementary experiment, we found that embryos transferred from GST to C-CPE at 5 h had either complete or caudal NTDs (Fig. 3A), while none of the embryos transferred from GST to C-CPE at 10 h exhibited NTDs. These data demonstrate that C-CPE-sensitive claudins are required for the phases of neural tube closure occurring between 5 h and 10 h in our culture system.

**Fig. 4:**
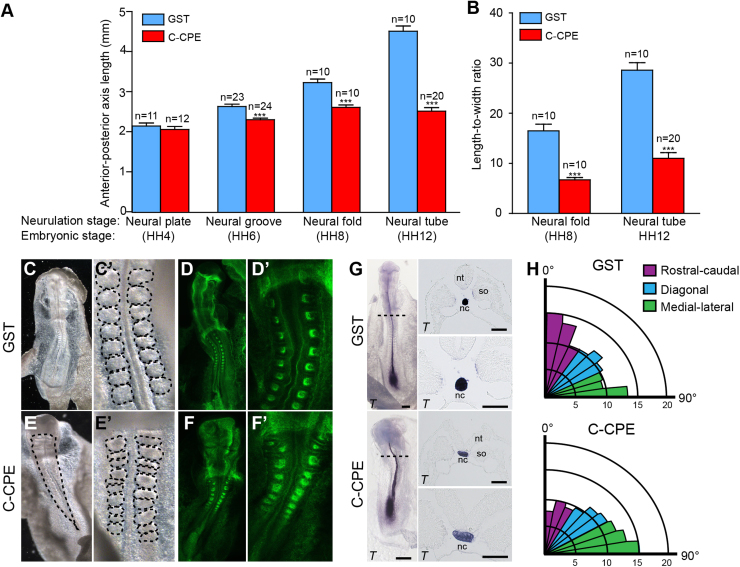
C-CPE-treated embryos exhibit convergent extension defects. **(A)** Graph shows average anterior-posterior (AP) length of GST- and C-CPE-treated embryos at different stages of neural tube closure (mean±s.e.m, ***p<0.0001, Mann-Whitney *U*-test). Corresponding Hamilton and Hamburger (HH) stages are indicated below. **(B)** Graph shows length-to-width ratio for GST- and C-CPE-treated embryos at neural fold and neural tube stages (mean±s.e.m., ***p<0.0001, Mann-Whitney *U*-test). **(C-F)** Dorsal views of embryos treated with 200 µg/ml GST (C, D) or C-CPE (E, F) for 20 h. Brightfield images (C,E) and Phalloidin-stained (D,F) embryos are shown. (C′-F′) Higher magnification images showing paired somites in GST-treated embryos (C′,D′) and unpaired and fused somites in C-CPE-treated embryos (E′,F′). **(G)** Left, dorsal view of *Brachyury* (*T*) expression following 20 h treatment with 200 µg/ml GST or C-CPE for 20 h. Scale bar, 0.5 mm. Right, transverse section corresponding to position indicated. Bottom images are higher magnification images of sections. Scale bar, 50 µm. nc, notochord; nt, neural tube; so, somite. **(H)** Rose diagram showing orientation of cell divisions relative to the midline in GST- (n=6 embryos/810 cells) and C-CPE-treated embryos (n=4 embryos/629 cells) at 5 h. Division angles are binned in bins of 10° from 0° to 90°. Purple bars represent rostrocaudal (RC) divisions, blue are mediolateral (ml) divisions, and green are diagonal (D) divisions. In GST-treated embryos, mitotic spindles are oriented preferentially in the RC plane compared to the ml plane (p=0.031) in C-CPE-treated embryos cell division was random (p=0.125) (Wilcoxon's signed-ranks test).

To identify the morphological events occurring between 5 h and 10 h, we incubated HH4 embryos with GST or C-CPE for 5 h, 10 h, or 20 h (Fig. 3B). At 5 h, GST-treated and C-CPE-treated embryos were HH6-8. In HH6 GST embryos, elongation of the neural plate and formation of the neural groove had begun and by HH8 the neural folds were elevated and met at the level of the midbrain (Fig. 3B). In contrast, C-CPE-treated HH6 embryos had broader neural grooves and their neural folds failed to meet at the level of the midbrain at HH8 (Fig. 3B). After 10 h of culture, the GST-treated control embryos were HH8^+^−10, had 5–10 pairs of somites and their neural folds were fused at the level of the brain and apposed at the anterior end of the spinal cord. Embryos cultured in C-CPE for 10 h were clearly distinguishable as their neural folds failed to converge towards the midline. After 20 h, GST-treated embryos had closed neural tubes, while all C-CPE-treated embryos had open neural tubes (Fig. 1D) and were significantly shorter than GST control embryos (Figs. 1D, 4A).

**Suppl. Fig. 5:**
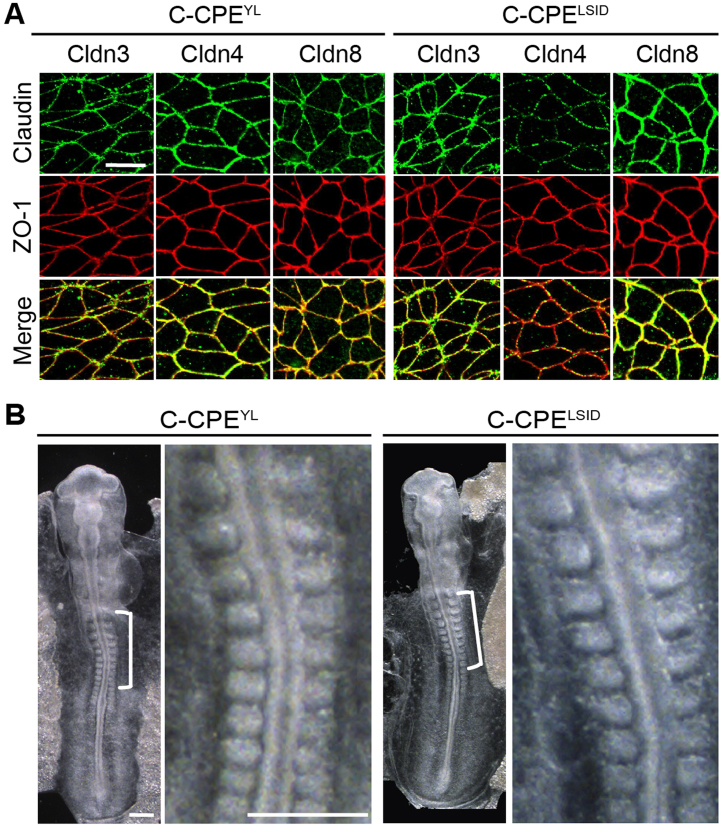
Cldn4-specific C-CPE variant, C-CPE^LSID^, does not cause NTDs. **(A)** Apical surface views of Cldn3, −4 or −8 (green) and ZO-1 (red) in embryos treated with C-CPE^YL^ or C-CPE^LSID^ for 5 h. Scale bar, 10 µm. Three embryos per treatment were analyzed. **(B)** Dorsal view of representative embryos incubated with C-CPE^YL^ (n=9) or C-CPE^LSID^ (n=12) for 20 h. Higher magnifications of bracketed region are shown to the right. Scale bar, 0.5 mm.

Cldn4 and −8 are the only C-CPE-sensitive claudins expressed in the neural ectoderm during these early phases. While we have successfully used morpholinos to knockdown expression of claudins in gastrulation-stage chick embryos (Collins et al., 2015), we and others have been unsuccessful at using this approach in neurulation-stage embryos (data not shown) (Colas and Schoenwolf, 2003). Therefore, we took advantage of a C-CPE variant that specifically targets Cldn4, C-CPE^LSID^ (Veshnyakova et al., 2012). C-CPE^LSID^ removed Cldn4 from tight junctions in the neural and non-neural ectoderm, had no effect on the localization of Cldn3 in non-neural ectoderm, and led to increased levels of Cldn8 at tight junctions in the neural ectoderm (Supplementary Fig. 5A). Embryos treated with C-CPE^LSID^ did not have NTDs (Supplementary Fig. 5B). Based on these data we hypothesize that either Cldn4 is not essential for neural tube closure or that the increased localization of Cldn8 to tight junctions compensates for the loss of Cldn4. Unfortunately, a C-CPE variant that specifically targets removal of Cldn8 from tight junctions does not exist and so we cannot distinguish between these two possibilities at the present time.

### 2.5 C-CPE-treated embryos have convergent extension and apical constriction defects

The morphology of the C-CPE-treated embryos was consistent with phenotypes caused by defective convergent extension movements (Lawson et al., 2001, Moury and Schoenwolf, 1995, Ybot-Gonzalez et al., 2007). First, C-CPE-treated embryos were shortened along their anterior-posterior axes (p<0.0001), wider (p<0.001) and displayed a reduced length-to-width ratio (p<0.001) at all stages of neurulation examined (Fig. 4A,B). Second, the somites of 20–40 h C-CPE-treated embryos were irregularly shaped and sometimes fused (Fig. 4C-F). Third, the notochord marker *Brachyury* (*T*) had a broadened posterior expression domain and the notochords were misshapen in 50% of the C-CPE-treated embryos (Fig. 4G). Finally, we observed an effect on oriented cell division, which helps shape the neural plate (Sausedo et al., 1997). In GST-treated embryos, mitotic spindles in the neural ectoderm were preferentially oriented rostrocaudally (p=0.031), while in C-CPE-treated embryos there was a decrease in the proportion of rostrocaudal divisions and corresponding increase in mediolateral divisions (p=0.125) (Fig. 4H). Together these data suggest that removal of C-CPE-sensitive claudins prevents normal convergent extension movements during neurulation, contributing to the increased distance between the neural folds thereby impeding neural fold fusion.

**Fig. 5:**
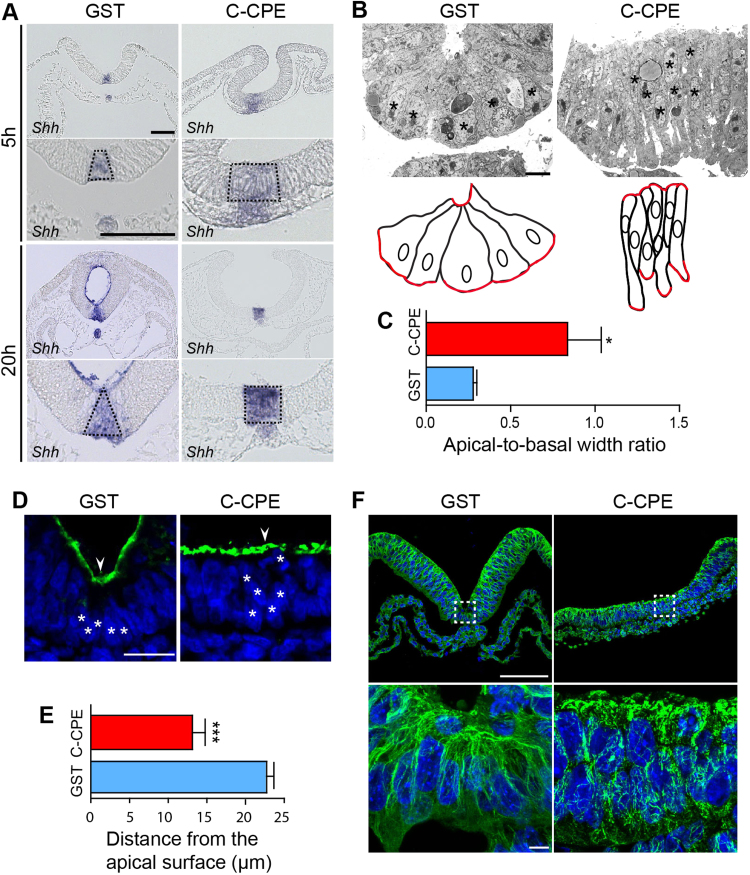
Neural plate midline cells are not apically constricted in C-CPE-treated embryos. **(A)** Transverse sections showing *Shh* expression in 5 h and 20 h GST- and C-CPE-treated embryos. Dashed line in higher magnification view marks *Shh* expression domain in floor plate. Scale bar, 50 µm. **(B)** Ultrathin sections through the neural tube of 20 h GST- and C-CPE-treated embryos processed for TEM. Nuclei are indicated by asterisks. Schematic representation is shown below. Apical and basal cell surfaces are outlined in red. **(C)** Graph illustrates apical-to-basal width ratio of median hinge point cells from embryos treated with GST (n=8 cells/2 embryos) or C-CPE (n=10 cells/2 embryos) (mean±s.e.m, *p =0.012), student *t*-test). Scale bar, 5 µm. **(D)** Transverse sections of 5 h GST- or C-CPE-treated embryos stained for Cldn1 (green) and DAPI (blue). Midline (white arrowhead) and nuclei (asterisks) are indicated. Scale bar, 10 µm. **(E)** Graph shows average distance of nuclei from apical cell surface in GST- (n=108 cells/2 embryos) and C-CPE-treated embryos (n=212 cells/3 embryos) (mean±s.e.m, ***p=0.0005, student *t*-test). **(F)** Top, transverse sections of 5 h GST- or C-CPE-treated embryos stained for α-tubulin (green) and DAPI (blue). Bottom, high magnification view of microtubules in the median hinge point of GST- and C-CPE-treated embryos. Scale bar, 100 µm.

The broadened neural groove in C-CPE-treated embryos suggested a failure in median hinge point formation. In GST-treated embryos, *Shh* exhibited a normal wedge-shaped expression domain at the neural plate midline reflecting the triangular shape of these cells (Fig. 5A). In C-CPE-treated embryos *Shh* expression was unaffected but the shape of its expression domain was rectangular rather than the triangular shape observed in control embryos (Fig. 5A). Indeed, transmission electron microscopy confirmed that midline neural tube cells in C-CPE-treated embryos were not apically constricted and triangular (Fig. 5B). The apical-to-basal width ratio of the midline cells was three times greater in C-CPE-treated embryos than in GST-treated embryos (p=0.012; Fig. 5C). Microtubule-dependent basal migration of the nuclei also contributes to the basal widening of the median hinge point cells (Smith and Schoenwolf, 1987, Karfunkel, 1971, Karfunkel, 1972). As expected, median hinge point cell nuclei were situated basally in GST-treated embryos (Fig. 5D). However, in C-CPE-treated embryos, these nuclei were randomly localized and on average closer to the apical surface than in GST-treated control embryos (p=0.0005) (Fig. 5D,E). In addition, the apical-basal microtubule network was discontinuous in C-CPE-treated embryos (Fig. 5F). Together these data demonstrate that C-CPE-sensitive claudins are required in the neural ectoderm for cell shape changes associated with median hinge point formation.

### 2.6 Claudins function upstream of PCP and RhoA/ROCK signaling

**Fig. 6:**
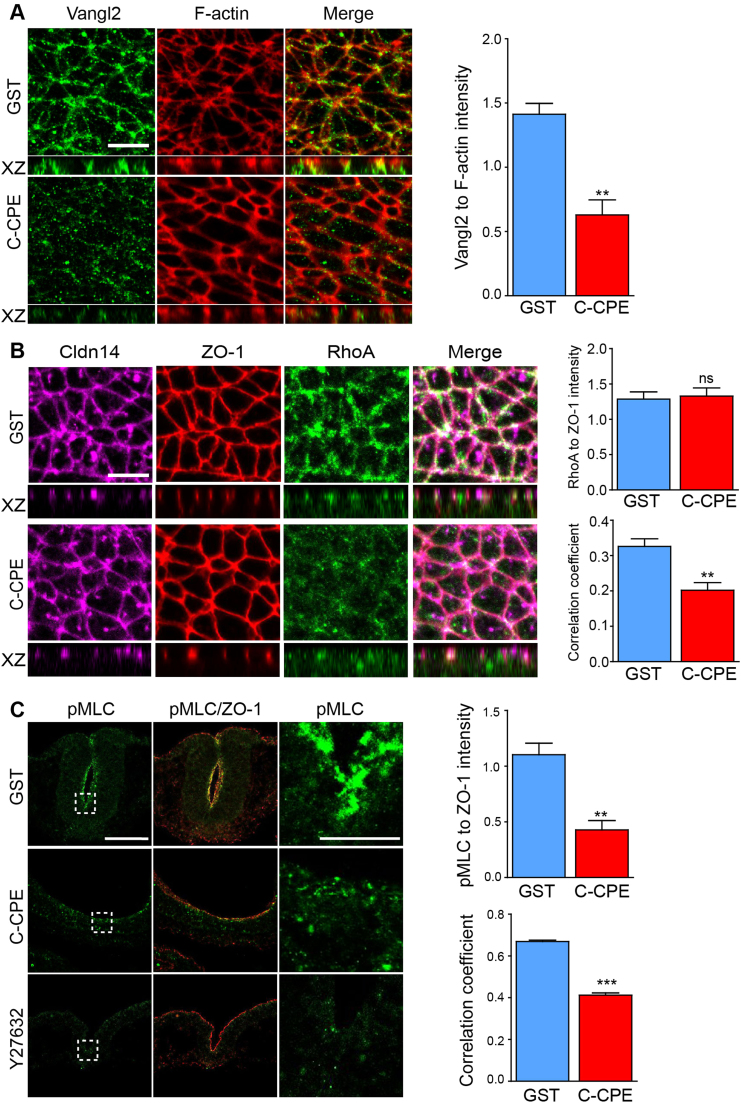
PCP and RhoGTPase signaling proteins are reduced at tight junctions in C-CPE-treated embryos. **(A)** Apical surface and orthogonal (XZ) views of Vangl2 (green) and F-actin (Phalloidin, red) in neural ectoderm of GST- or C-CPE-treated embryos at 5 h. Scale bar, 10 µm. Graph shows the ratio of the immunofluorescence intensity of Vangl2 to F-actin. Histogram represents an average of three embryos and values were calculated from maximum intensity projections (mean±s.e.m, **p=0.0024, student *t*-test). **(B)** Apical surface and orthogonal (XZ) views of Cldn14 (pink), ZO-1 (red), and RhoA (green) in the neural ectoderm of GST or C-CPE-treated embryos at 5 h. Scale bar, 10 µm. Graphs show the ratio of the immunofluorescence intensity of RhoA to ZO-1 (upper), and Pearson's correlation coefficient for RhoA and ZO-1 (lower). Histograms represent an average of three different fields from three embryos and values were calculated from maximum intensity projections (mean±s.e.m, ns = not significant, **p=0.0052, student *t*-test). **(C)** Transverse sections of embryos treated with GST, C-CPE or Y27632 for 5 h showing pMLC (green) and ZO-1 (red). Scale bar, 100 µm (left and middle) and 10 µm (right). Graphs show the ratio of the immunofluorescence intensity of pMLC to ZO-1 (upper), and Pearson's correlation coefficient for pMLC and ZO-1 (lower). Data were collected from the enclosed areas of sectioned neural plates. Histograms represent an average of three embryos and values were calculated from maximum intensity projections (mean±s.e.m, **p=0.0023, ***p<0.0001, student *t*-test).

**Suppl. Fig. 6:**
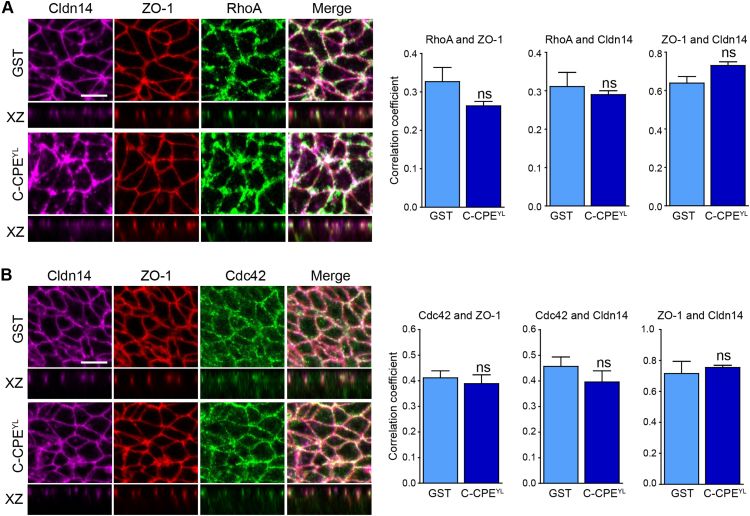
Localization of Cldn14, RhoA, and Cdc42 is not affected in embryos treated with C-CPE^YL^. **(A)** Confocal views of triple-immunostaining for Cldn14, ZO-1 and RhoA at the apical surface of neural ectoderm of embryos treated with GST or C-CPE^YL^ for 5 h. The bar graphs show Pearson's correlation coefficient for RhoA and ZO-1 (p=0.182), RhoA and Cldn14 (p=0.608) or ZO-1 and Cldn14 (p=0.08). Histograms are the average of three different fields from three embryos (mean±s.e.m, student *t*-test). **(B)** Confocal views of triple-immunostaining for Cldn14, ZO-1 and Cdc42 at the apical surface of neural ectoderm of embryos treated with GST or C-CPE^YL^ for 5 h. The bar graphs show Pearson's correlation coefficient for Cdc42 and ZO-1 (P=0.642), Cdc42 and Cldn14 (P=0.344) or ZO-1 and Cldn14 (P=0.652) (mean±s.e.m, student *t*-test).

**Suppl Fig. 7:**
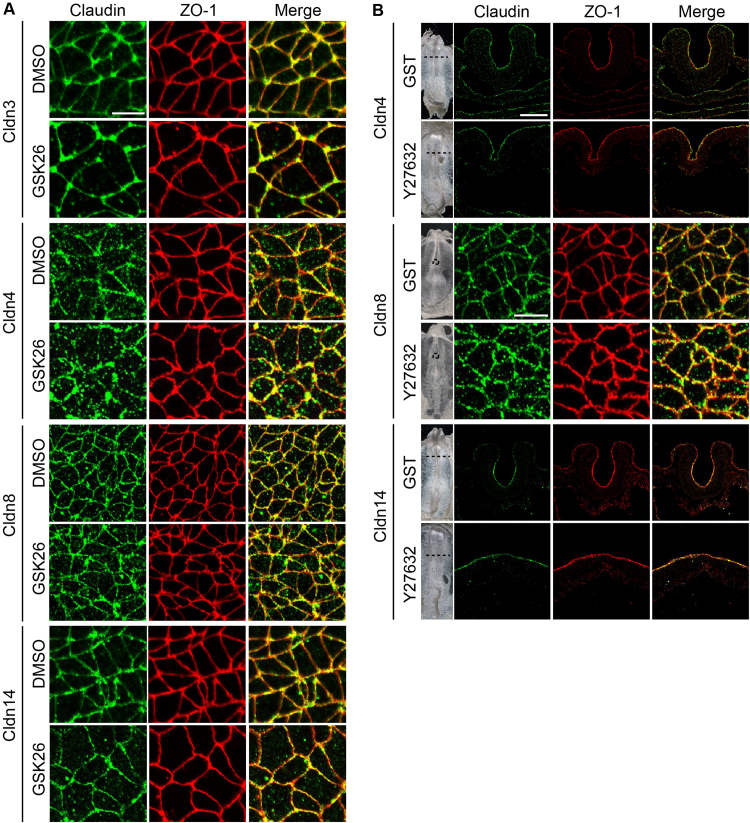
Inhibitors of ROCK signaling did not affect Claudin expression or localization. **(A)** Apical surface views of ZO-1 (red) and Cldn3, −4, −8 and −14 (green) expression in embryos incubated in DMSO (control) or GSK269962 for 5 h. **(B)** Left, dorsal views of neural fold stage embryos treated with GST or Y27632 for 5 h. Transverse sections showing Cldn4 or −14 (green) and ZO-1 (red) expression and apical surface view of an embryo co-stained for Cldn8 (green) and ZO-1 (red) expression.

Convergent extension and apical constriction during neurulation require the coordinated activities of the PCP and RhoA/ROCK signaling pathways, respectively (Nishimura et al., 2012; Kinoshita et al., 2008; Ossipova et al., 2015). In order to position claudins in the context of these pathways, we examined the expression of Vangl2, a core component of the PCP pathway that is required for neural tube closure (Kibar et al., 2011, Murdoch et al., 2001), RhoA, a small GTPase that shuttles between the cytoplasm and membrane, and phosphorylated myosin light chain (pMLC), the downstream target of RhoA/ROCK signaling. Vangl2 was greatly reduced after 5 h of C-CPE-treatment (p=0.0024) (Fig. 6A). RhoA partly co-localized with ZO-1 and Cldn14 at the membrane in the neural ectoderm of GST-treated embryos and its localization was unchanged in C-CPE^YL^-treated embryos (Fig. 6B, Supplementary Fig. 6A). However, after 5 h of C-CPE-treatment, we observed the loss of RhoA from tight junctions and a significant reduction in phosphorylated myosin light chain (pMLC) in the neural ectoderm of C-CPE-treated embryos (Fig. 6B,C). Quantitative analysis revealed that although the total amount of RhoA did not change (p=0.7882), the correlation between RhoA and ZO-1 in C-CPE-treated embryos was ~40% that of GST-treated embryos (p=0.0052). Both the total level of pMLC (p=0.0023) and its distribution to tight junctions (p<0.0001) were significantly reduced in C-CPE-treated embryos. Incubating embryos with the ROCK inhibitors GSK 269962 and Y-27632 did not affect the expression or localization of Cldn3, −4 or −8 (Supplementary Fig. 7), although they did reduce the level of pMLC and cause NTDs as previously reported (Fig. 6C) (Kinoshita et al., 2008, Escuin et al., 2015). These data further support that claudins function upstream of PCP and Rho/ROCK signaling during neural tube closure.

**Fig. 7:**
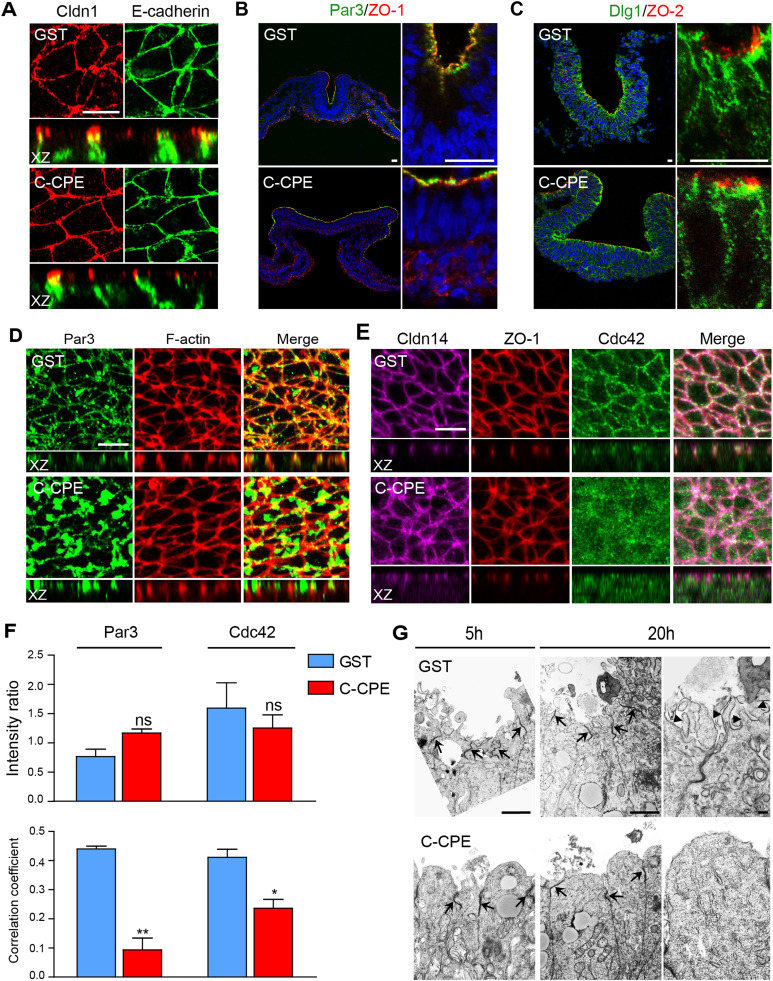
C-CPE-sensitive claudins are required for Par3 and Cdc42 localization to the lateral membranes at the apical cell surface. (A) Apical surface and XZ orthogonal views of Cldn1 (red) and E-cadherin (green) in 5 h GST- and C-CPE-treated embryos. Scale bar, 20 µm. (B) Transverse section from 5 h GST- and C-CPE-treated embryos showing Par3 (green) and ZO-1 (red). Scale bar, 20 µm. (C) Transverse section from 5 h GST- and C-CPE-treated showing ZO-2 (red) and Dlg1 (green). Scale bar, 10 µm. (D) Apical surface and XZ orthogonal views of Par3 (green) and F-actin (Phalloidin, red) in the neural ectoderm of 5 h GST- or C-CPE-treated embryos. Scale bar, 10 µm. (E) Apical surface and XZ orthogonal views of Cldn14 (pink), ZO-1 (red) and Cdc42 (green) in the neural ectoderm of 5 h GST- or C-CPE-treated embryos. Scale bar, 10 µm. Three embryos per treatment were analyzed. (F) Graphs show the ratio of the immunofluorescence intensity of Par3 to F-actin or Cdc42 to ZO-1 (upper), and Pearson's correlation coefficient for Par3 and F-actin or Cdc42 and ZO-1 (lower). Histograms represent an average of three different fields from three embryos and values were calculated from maximum intensity projections (mean±s.e.m, ns = not significant, *p=0.0132, **p=0.0072, student *t*-test). (G) TEM micrographs of transverse sections at the neural tube midline of 5 h and 20 h GST- and C-CPE-treated embryos. Arrows indicate tight junctions and arrowheads indicate microvilli. Scale bar, 500 nm.

Analysis of apical-basal polarity suggested that the decreased apical localization of Vangl2, RhoA and pMLC in C-CPE-treated embryos was not due to effects on the establishment or maintenance of apical-basal polarity. Cldn1, Cldn14, ZO-1 and 2, and F-actin were apically localized in C-CPE-treated embryos (Figs. 1, 5D and 6). In both GST- and C-CPE-treated embryos Cldn1 was localized apical to the adherens junction protein E-cadherin (Fig. 7A), Par3 co-localized with ZO-1 (Fig. 7B) and ZO-2 localized apical to the Scribble complex protein Dlg1 (Fig. 7C). These data suggest that localization and/or retention of Vangl2, RhoA and pMLC to the apical membrane is dependent on the presence of C-CPE-sensitive claudins in the neural tube.

### 2.7 C-CPE treatment affects cell morphology and protein localization at the apical surface

Apical surface views showed that claudins, ZO scaffolding proteins, E-cadherin and F-actin localized normally to the lateral cell membranes in C-CPE-treated embryos (Figs. 2B and C, 6A and B and 7A). This was not the case for Par3; although restricted to the apical domain, large aggregates of Par3 were present in the cytoplasm (Fig. 7D). Similarly, the RhoGTPase Cdc42 was observed across the apical surface and less enriched at lateral membranes as compared to embryos treated with GST or C-CPE^YL^ (Fig. 7E, Supplementary Fig. 6B). Consistent with our visual analysis, we found that the total amount of Par3 (p=0.107) and Cdc42 (p=0.523) was not affected by C-CPE treatment, but that their distribution was altered (p<0.05) (Fig. 7F). These data also support unique roles for individual claudins in maintaining protein complexes at the apical tight junction cytoplasmic plaque.

TEM analysis of tight junction ultrastructure revealed that electron dense tight junctions between cells at the neural plate midline in C-CPE-treated embryos were indistinguishable from those in GST-treated embryos (Fig. 7G). However, in C-CPE-treated embryos the apical surface of these cells was smooth, convex and devoid of microvilli (Fig. 7G) as compared to cells in GST-treated embryos, which contained numerous microvilli. Changes in morphology of the apical cell surface may be secondary to the altered localization of Cdc42, which is a critical regulator of the microtubule network. Together these data revealed roles for C-CPE-sensitive claudins in regulating the shape of the apical cell surface.

## 3 Discussion

Claudins are known to have distinct roles in determining cell shape (Wu et al., 2004, Nelson et al., 2010), maintaining epithelial integrity (Ikenouchi et al., 2003) and embryonic morphogenesis (Siddiqui et al., 2010). In this study, we demonstrated for the first time that claudins have a direct role in neural tube closure: C-CPE removal of Cldn3, −4 and −8 from tight junctions in the neural and non-neural ectoderm resulted in NTDs in chick and mouse embryos. Although disrupting ectoderm differentiation can affect neural tube closure (Kimura-Yoshida et al., 2015, Ybot-Gonzalez et al., 2007), gene expression analyses support that this is not the underlying cause of the C-CPE-induced NTDs. However, PCP and Par complex polarity proteins, and the small GTPases RhoA and Cdc42 were inappropriately localized at the apical cell surface in C-CPE-treated chick embryos. Consequently, convergent extension and apical constriction, two critical morphogenetic events that drive neural tube closure, were disrupted. Folic acid supplementation was unable to rescue the open NTDs in C-CPE-treated chick embryos suggesting that claudin-mediated morphogenetic events underlie at least a subset of NTDs not prevented by folate dietary supplementation and fortification.

### 3.1 Claudins in convergent extension movements

Convergent extension, which coordinates anterior-posterior lengthening and mediolateral narrowing of the neural plate, is dependent on PCP signaling. C-CPE-treated embryos exhibited hallmarks of convergent extension defects similar to those observed in the PCP mouse mutants *Lp/Lp* and *Dvl1*^*–/–*^;*Dvl2*^*–/–*^ (Ybot-Gonzalez et al., 2007; Wang et al., 2006), suggesting that claudins and PCP signaling function in the same molecular pathway. The reduced expression of the core PCP protein Vangl2 in C-CPE-treated embryos positions claudins upstream of the PCP pathway in directing convergent extension cell movements. Furthermore, Vangl2 has been implicated in regulating anterior-posterior cell divisions during zebrafish neurulation (Ciruna et al., 2006) and we observed similar defects in oriented cell division in C-CPE-treated embryos. Therefore, we speculate that the loss of Vangl2 is responsible for the oriented cell division defects observed in these embryos. However, we cannot rule out the possibility that this is caused by disrupted localization of Par3 (Tawk et al., 2007) and Cdc42 (Kieserman and Wallingford, 2009) as they also play a role in oriented cell division. While a role for PCP-dependent convergent extension in avian neural tube closure has not been assessed, siRNA-mediated knockdown of the PCP component Celsr1 causes NTDs in chick embryos (Nishimura et al., 2012). This observation, along with the fact that overexpression and knockdown of PCP components causes convergent extension defects during neurulation in *Xenopus* (Goto and Keller, 2002; Wallingford and Harland, 2002) and zebrafish (Tawk et al., 2007) embryos, argues for a conserved role of the PCP pathway in regulating convergent extension during neural tube closure. Convergent extension defects were not observed in C-CPE-treated mouse embryos because C-CPE treatment was initiated at E8.5 after convergent extension is underway and neural tube closure has initiated. Interestingly, caudal elongation defects were observed in mouse embryos that were treated at the 0–4 somite stage (EN and NDEG, unpublished observations).

C-CPE removed claudins from the neural (Cldn4 and −8) and non-neural ectoderm (Cldn3 and −4), both of which undergo convergent extension movements. Decreased expression of *Cldn3*, *−4*, *−6* and *−7* in the non-neural ectoderm is also observed in *Grhl2* mutant mice, which have folate-resistant NTDs. Grainyhead-like 2 (Grhl2) is a transcription factor that is expressed exclusively in the non-neural ectoderm where it regulates expression of genes involved in epithelial identity, including claudins (Brouns et al., 2011; Pyrgaki et al., 2011, Werth et al., 2010). Although the neural folds remain widely spaced in the *Grhl2* mutants, convergent extension and apical constriction occur normally (Pyrgaki et al., 2011, Rifat et al., 2010, Werth et al., 2010). These data support our hypothesis that the convergent extension and apical constriction defects observed in C-CPE-treated embryos are due to the depletion of C-CPE-sensitive claudins from the neural ectoderm.

### 3.2 Claudins function upstream of RhoGTPase signaling during neural tube closure

Morphological, molecular and ultrastructure analysis of C-CPE-treated embryos revealed cells in the neural plate midline were not apically constricted and consequently the median hinge point did not form. Establishment of the apical membrane domain in epithelial cells depends on the coordination of the intracellular trafficking machinery, RhoGTPase signaling and the Par3-Par6-aPKC polarity complex. Despite a clear demarcation of the apical and basolateral domains in C-CPE-treated embryos, Par3 was abnormally localized in large aggregates. Knockdown of Par3 in HH12 chick embryos, following neural tube closure, results in closed NTDs (Chen et al., 2017). Our data suggest that Par3 also plays a role during earlier stages of neurulation in avian embryos. We presume that the aggregated Par3 either cannot fulfill its normal function and/or interferes with other protein trafficking at the apical surface.

During neural tube closure, apical localization of RhoA-ROCK signaling components at the neural plate midline is required for phosphorylation of myosin light chain, which then moves along actin filaments to generate the contractile force required for apical constriction (Kinoshita et al., 2008, van Straaten, 2002). Chick embryos treated with C3, an inhibitor of Rho GTPases, develop NTDs (Kinoshita et al., 2008). Similarly, inhibiting Rho kinase (by Y27632) or myosin II motor activity (by blebbistatin) causes open NTDs in chick (Kinoshita et al., 2008) and mouse embryos (Escuin et al., 2015). In C-CPE-treated embryos, junctional localization of RhoA was reduced leading to reduced levels of pMLC. Treatment with ROCK inhibitors, which significantly decreased the level of pMLC and caused open NTDs (Kinoshita et al., 2008, Escuin et al., 2015), did not affect claudin expression or localization. These data indicate that claudins function upstream of RhoA-ROCK signaling in the neural ectoderm during neural tube closure and that recruitment and retention of RhoA at the cytoplasmic plaque is dependent on direct or indirect interactions with claudins expressed in the neural ectoderm.

RhoA signaling is required for changing the claudin composition of tight junctions via endocytosis (Quiros and Nusrat, 2014). In *Xenopus* embryos endocytosis acts downstream of actin-myosin contraction to remove excess membrane from the apical surface; inhibiting endocytosis leads to neural tube defects due to defective apical constriction (Lee and Harland, 2010). Currently there is no evidence to suggest that endocytosis is required for apical constriction of neural ectoderm cells in the chick embryo. However, it is tempting to speculate that RhoA interactions with Cldn8 and/or Cldn4 contribute to remodeling of the apical cell surface in the chick neural ectoderm via endocytosis.

Actin-myosin-dependent apical constriction in midline neural plate cells occurs in concert with apical-basal shortening and basal nuclear migration, which are dependent on an apically-basally aligned microtubule network. Nuclear migration was disrupted in C-CPE-treated embryos and Cdc42, a critical regulator of the microtubule network (Palazzo et al., 2001, Cadot et al., 2012), was expressed diffusely across the apical surface rather than being enriched at the lateral membrane. We hypothesize that the altered localization of Cdc42 affects microtubule-dependent events during median hinge point formation. Defects in the microtubule cytoskeleton may also underlie the altered shape of the apical surface of midline cells in C-CPE-treated embryos, which were smooth, convex and devoid of the microvilli present in control embryos. Our data indicate that Cldn8 and/or Cldn4, C-CPE-sensitive claudins expressed in the neural ectoderm, uniquely contribute to the retention of Par polarity complex proteins and Cdc42 at the lateral cell membrane at the neural plate midline.

In conclusion, we have defined new roles for C-CPE-sensitive claudins in regulating cell movements and shape changes that are critical for neural tube morphogenesis. Our data support a model where disturbing the claudin composition of tight junctions impacts protein complexes at the cytoplasmic plaque. Previous studies have suggested that Vangl2 polarization and Rho-induced phosphorylation of myosin light chain participate in a mutually dependent feedback loop (Nishimura et al., 2012, Ossipova et al., 2015). Our data suggest that claudin function intersects with this pathway to regulate Vangl2 localization to the apical cell surface and phosphorylation of myosin light chain during convergent extension and apical constriction, respectively. Both of these events are intrinsic to the neural ectoderm, where Cldn4 and −8 are the only C-CPE-sensitive claudins, *Cldn4* null mice have closed neural tubes (Fujita et al., 2012) and *Cldn8* null mice have not yet been reported. In the mouse kidney, Cldn4 and −8 are known to interact and Cldn8 is required for recruitment of Cldn4 to the tight junction (Gong et al., 2015, Hou et al., 2010). Therefore, we hypothesize that Cldn8 collaborates with Cldn4 to participate in unique interactions with signaling complexes at the tight junction cytoplasmic plaque, which regulate the morphogenetic movements and cell shape changes required for neural plate elongation and median hinge point formation. Claudin C-terminal domains are highly variable between family members and are uniquely regulated by phosphorylation and other post-translational modifications, which can influence their interactions with proteins in the tight junction cytoplasmic plaque (Findley and Koval, 2009). While individual claudins may not be required to maintain the apical domain or tight junction integrity, they are likely to each have unique roles in regulating intracellular events that affect the actin cytoskeleton and cell behavior, potentially in a cell-type or tissue-specific manner. Future studies will be needed to understand how claudins anchor these proteins to the apical cell surface, to determine if these interactions are direct or indirect, and how they are influenced by post-translational modifications.

Few of the more than 200 NTD mouse models have led to the identification of genetic mutations associated with human NTDs (Harris and Juriloff 2010, Kibar et al., 2007). This is likely due to the complex etiology of the disease which results from gene-environment and gene-gene interactions. While single claudin knock-out mice do not exhibit NTDs, we showed that simultaneous removal of Cldn3, −4 and −8 from tight junctions results in NTDs that parallel the phenotypically diverse NTDs that can result from a common underlying gene defect in humans (Lei et al., 2013, Robinson et al., 2012, Juriloff and Harris, 2012). There are numerous examples of diseases where birth defects result from mutations in multiple genes that act synergistically (Kim et al., 2016, Nanni et al., 1999, Kajiwara et al., 1994, Katsanis et al., 2001). While there has been limited success in identifying mutations in the human orthologues of mouse candidate NTD genes, an approach that looks at multiple genes that interact in a signaling pathway may be more successful. Our discovery of roles for C-CPE-sensitive claudins in neural tube closure, and their intersection with pathways known to be involved in neural tube closure, including RhoGTPase and PCP signaling, suggests that the claudin family represents new candidates for NTDs in humans.

## 4 Materials and methods

### 4.1 Production of GST and GST-C-CPE fusion protein

Expression of GST, GST fused N-terminally to the C-terminal amino acids 185–319 (Moriwaki et al., 2007) and the variants C-CPE^LSID^ (L254A/S256A/I258A/D284A) and C-CPE^YL^ (Y306A/L315A) cloned into pET14b or pGEX6P1 was induced by IPTG. GST fusion proteins were purified from *E. coli* BL21 as described previously (Protze et al., 2015, Veshnyakova et al., 2012) and dialyzed against PBS. Protein concentration was determined using the BCA Protein Kit (Thermo-Scientific, Rockford, USA) or Bio-Rad Protein Assay (BioRad, Mississauga, Canada).

### 4.2 Embryo culture

Fertilized eggs (Couvoir Simetin, Mirabel, Canada) were incubated at 38.5 °C. Embryos were collected, staged according to Hamilton and Hamburger (HH) criteria (Hamburger and Hamilton, 1951) and cultured using the modified Cornish pasty method (Nagai et al., 2011). Purified GST or C-CPE (50–500 µg/ml), ROCK inhibitors Y27632 (50 μM; Abcam, Cambridge, UK) or GSK 269962 (1 μM; Axon Medchem, Reston, USA), and folic acid (100 µM or 1 mM) were added directly to the Cornish pasty culture medium to achieve the final concentrations indicated.

E8.5 mouse embryos (E0.5 is noon on the day after overnight mating) were dissected in Dulbecco's Modified Eagle's Medium containing 10% fetal calf serum. The yolk sac and amnion were left intact for whole embryo culture (Cockroft, 1990, Pryor et al., 2012). GST or C-CPE were diluted with PBS. Fast Green (0.5% solution) was added to visualize injection. Microinjection was performed using a hand-held glass micropipette. Needle tips were positioned in the amniotic cavity by traversing the yolk sac and amnion and 1–2 µl were injected, as previously described (Abdul-Aziz et al., 2009). Following 18 h of culture, embryos were examined. Yolk sac circulation was used as an indicator of viability, somites were counted as a measure of developmental progression, and length of the posterior neuropore was measured using an eyepiece graticule.

### 4.3 Immunofluorescence staining

Immunostaining was performed on paraffin sections (for α-tubulin), cryosections or whole embryos fixed in 4% PFA or 10% trichloroacetic acid at 4 °C. Samples were incubated overnight at 4 °C with primary antibodies: Cldn1, −4 and −8 (Invitrogen, Carlsbad, USA, 1:25–1:50), Cldn3 (Abcam, Cambridge, UK, 1:50), Cldn14 (Sigma Aldrich, Oakville, Canada, 1:25), ZO-1 and ZO-2 (Invitrogen, 1:50, Carlsbad, USA), anti-disphospho-myosin light chain (Thr18/Ser19) (Cell Signaling, Ipswich, USA, 1:50), RhoA and Cdc42 (Santa Cruz, Santa Cruz, USA, 1:50), Par3 (Millipore, Etobicoke, Canada, 1:250), Dlg1 (US Biologicals, Salem, USA, 1:50), E-cadherin (BD Transduction, San Jose, USA, 1:100), α-tubulin (Abcam, Cambridge, UK, 1:100) or Vangl2 (gift from Dr. M Montcouquiol, 1:500). Alexa Fluor-conjugated secondary antibodies (1:500) were added for 1 h at RT. F-actin was detected using Alexa Fluor-conjugated Phalloidin (Molecular Probes, Eugene, USA). Sections and flat-mounted embryos were coverslipped with SlowFade Gold with DAPI (Molecular Probes, Eugene, USA) and imaged using a Zeiss LSM780 laser scanning confocal microscope. Colocalization and immunoquantification was performed using ZEN 2012 SP1 software (Carl Zeiss Microscopy, Germany).

### 4.4 Whole mount *in situ* hybridization

Antisense digoxygenin-labeled riboprobes for *Sox2*, *AP-2α*, *Pax7* (Khudyakov and Bronner-Fraser, 2009), *Otx2* (Chapman et al., 2002, Chapman et al., 2003), *Shh*, *Brachyury*, and *Pax6* were used as previously described (Collins and Ryan, 2011). Embryos were photographed using a Leica M125 dissecting microscope with Infinity Capture software v5.0.2 (Lumenera Corp.). Paraffin-embedded embryos were sectioned and photographed using a Zeiss Axiophot compound microscope and AxioCamMRc camera with Axiovision v4.7.1.0 software.

### 4.5 Transmission electron microscopy

Specimens were prepared at the Facility for Electron Microscopy Research, McGill University. Embryos were fixed with 2.5% glutaraldehyde, 0.1% calcium chloride and 4% sucrose in 0.1 M sodium cacodylate buffer overnight at 4 °C, post-fixed with 1% aqueous osmium and 1.5% potassium ferrocyanide in 0.1 M sodium cacodylate for 2 h at 4 °C, dehydrated with a graded series of acetone, and embedded in epon. 100 nm sections were cut using an UltraCutE ultramicrotome (Reichert-Jung) and stained with uranyl acetate and Reynold's lead. Images were acquired on an FEI Techai 12 120 kV transmission electron microscope equipped with an AMT xR80C 8 megapixel CCD camera.

### 4.6 Morphometric assessment of axial length, length-to-width ratios, apical constriction and oriented cell division

Dorsal images of embryos were photographed using a Leica M125 dissecting microscope with Infinity Capture software v5.0.2 (Lumenera Corp.) and imported into SPOT Advanced image capture software (SPOT Imaging Solutions). Width of the neural folds or neural tube at the level of the first somite and anterior-posterior length were measured. To assess apical constriction, individual cells of the median hinge point were examined in transverse ultrathin sections processed for transmission electron microscopy from embryos treated for 20 h with GST or C-CPE. Cell surfaces were measured using ImageJ software. Basal nuclear migration was determined by drawing a perpendicular line from the apical surface to the apical tip of each DAPI-positive nucleus in GST- and C-CPE-treated embryos at 5 h. To quantify the orientation of cell division, z-stacks of embryos stained with DAPI were exported into ImageJ. A line was drawn between the mitotic spindles of anaphase cells and the angle between this axis and the midline of embryos was measured. The angles of divisions were then exported to Microsoft Excel and converted into a 0° to 90° range plot as previously described (Shafer et al., 2017). For statistical purposes, spindle orientation measurements were separated into three bins: 0–30° (rostral-caudal), 30–60° (diagonal) or 60–90° (medial-lateral).

### 4.7 Whole mount TUNEL assay

GST- or C-CPE-treated embryos (200 µg/ml) were fixed with 4% PFA overnight at 4 °C and assessed using the *in situ* cell death detection kit, AP (Roche, Montreal, Canada). Neural and non-neural ectoderm of each embryo was imaged at the level of the hindbrain and first and last somites. Cell death index was calculated by dividing the number of TUNEL-positive nuclei by the total number of cells, as determined by Phalloidin staining in each 125 µm by 125 µm region.

### 4.8 Statistical analyses

Statistical significance was evaluated using the Mann-Whitney *U*-test, student *t*-test or Wilcoxon's signed-rank test through GraphPad InStat software or SigmaStat v.2 (SPSS Inc).

## Competing interests

The authors have no competing interests.
